# Types and Distribution of Organic Amines in Organic Nitrogen Deposition in Strategic Water Sources

**DOI:** 10.3390/ijerph19074151

**Published:** 2022-03-31

**Authors:** Yixuan Yang, Tongqian Zhao, Huazhe Jiao, Li Wu, Chunyan Xiao, Xiaoming Guo

**Affiliations:** 1 Institute of Resources and Environment, Henan Polytechnic University, Jiaozuo 454000, China; yangyixuan@hpu.edu.cn (Y.Y.); wuli@hpu.edu.cn (L.W.); xiaochunyan@hpu.edu.cn (C.X.); guoxiaoming@hpu.edu.cn (X.G.); 2 School of Civil Engineering, Henan Polytechnic University, Jiaozuo 454003, China; jiaohuazhe@hpu.edu.cn

**Keywords:** atmospheric nitrogen deposition, strategic water sources, organic nitrogen, organic amines

## Abstract

Organic nitrogen (ON) is an important part of atmospheric nitrogen deposition, but the content and distribution of components other than urea and amino acids are the blind area of current research. The deposition of organic amines (OA) in strategic water sources poses a great public health risk to unspecified populations. In order to further reveal the composition of about 50% soluble organic nitrogen, besides urea and amino acids, five functional sampling points (such as industrial area, agricultural area, urban area, tourism area and forest area) were set in the reservoir area to detect dissolved total nitrogen (DTN), dissolved organic nitrogen (DON) and OA components. The results show that the total nitrogen concentration was 6.42–10.82 mg/m^3^ and the DON concentration was 2.77–4.99 mg/m^3^. Ten kinds of OA were detected: dimethylamine (DMA), diethylamine (DEA), propylamine (PA), butylamine (BA), pyrrolidine (PYR), dibutylamine (DBA), N-methylaniline (NMA), 2-ethylaniline (2-ELA), benzylamine (BMA), and 4-ethylaniline (4-ELA). The average concentrations were 7.64, 26.35, 14.51, 14.10, 18.55, 7.92, 10.56, 12.84, 13.46 and 21.00 ng/m^3^, respectively. The total concentration of ten OA accounted for 2.28–9.81% of DON in the current month, of which the content of DEA was the highest, reaching 0.71%, the content of 4-ELA, PYR, PA and BA was 0.4–0.56%, and the content of DMA, DBA and NMA was 0.2–0.36%. The sources of OA in the reservoir area have significant seasonal differences. The content is the highest in spring, followed by autumn, and lower in summer and winter. The rainfall in spring and autumn is small, the source of road dust is relatively high, and the rainfall in summer is large. After the particles in the air are washed by rain, the concentration of OA in the sample is the lowest. On account of spring and autumn being the time of frequent agricultural activities, the concentration of OA is significantly higher than that in winter and summer.

## 1. Introduction

The effects of nitrogen deposition on ecological environment have multiple linkage and amplification effects [1]. Proper nitrogen deposition contributes to the improvement of ecosystem productivity, while excessive nitrogen deposition will have a significant impact on ecosystem health, such as water eutrophication, loss of biodiversity, leaching of soil nutrient elements, etc. [2]. At present, scholars in China and abroad have carried out a series of studies on nitrogen deposition flux, deposition characteristics and ecological effects. Among them, the research on terrestrial ecosystem is mainly focused on forests [3], grasslands [4], farmland [5], regions [6] and cities [7], and the research on nitrogen deposition in water is mainly focused on oceans [8], lakes [9,10] and swamps [11]. At present, most studies focus on inorganic nitrogen and ignore organic nitrogen, which leads to the general underestimation of the total flux of nitrogen deposition, resulting in the underestimation of the risk of nitrogen deposition in the ecosystem [12].

Organic nitrogen (ON) is an important part of nitrogen deposition, and organic nitrogen accounts for about 15–30% of the total deposition flux [13]. In the existing studies, the organic nitrogen component that can be quantified is less than 50% of the total organic nitrogen. Although researchers have carried out a lot of work, the composition of organic nitrogen is still unknown to a great extent, and there are many knowledge gaps. There are few reports on the deposition of organic amine nitrogen in reservoirs.

Organic amines (OAs) are a kind of trace compound that exist widely in the atmosphere and are one of the most important nitrogen-containing organic compounds [14]. At present, about 150 kinds of OA have been identified in the atmosphere, mainly including 24 kinds of methylamine, DMA, trimethylamine, ethylamine, diethylamine, PA and aniline [15]. OAs usually have a low olfactory threshold. When they reach a certain concentration in the air, they are not only disgusting in smell, but also cause damage to human health [16]. China stipulates that the allowable mass concentrations of methylamine, DMA and trimethylamine in the air are 5 μg/m^3^, 10 μg/m^3^ and 5 μg/m^3^, respectively [17]. OAs are mainly divided into fatty amines and aromatic amines. It is found that the exposed fatty amines enter the human body through inhalation, intake, or skin absorption, which will cause damage to the eyes, skin, liver, kidney, respiratory system, cardiovascular system and central nervous system, but most fatty amines will not cause cancer [18]. Some aromatic amines (such as aniline, benzidine and aminobiphenyl) were found to be carcinogens, and the mass concentration reaches 415.1 mg/m^3^ aniline, which can cause immediate death [19].

OAs come from a wide range of sources, which can be generally divided into natural sources and man-made sources. Natural sources mainly include biomass combustion [20], marine sources [21], soil sources and plant emissions, while human sources mainly include livestock emissions, industrial sources, automobile exhaust, fertilizer use [22] and human activities [23]. The existing forms of OA in the atmosphere are mainly divided into gaseous and granular forms [24]. Studies have shown that due to the high water solubility of OA, it is possible for OA to exist in aqueous aerosols [25]. The active nitrogen emitted from emission sources will affect the ecosystem through atmospheric deposition. Pan et al. [26] set up 10 stations in northern China and conducted field observation for 3 years to study nitrogen deposition. The results show that the level of nitrogen deposition in northern China is high, and the annual average flux is 6.06 × 10^3^ kg·km^−2^·a^−1^. Granular nitrogen accounts for 10% of the total flux of nitrogen deposition, and oxidizing substances account for 21% of the total flux, suggesting that other forms of gaseous nitrogen such as ammonia account for the dominant part of the total flux of nitrogen deposition. Han et al. [27] continuously measured atmospheric ammonia in Xi’an, and obtained that the annual average mass concentrations of ammonia in urban and suburban areas of Xi’an were 12.9 μg/m^3^ and 14.1 μg/m^3^, respectively, showing a trend of summer > spring > autumn > winter. Because there are few research results on atmospheric OA, the existing data are not enough to estimate the national flux, especially various aromatic amines that are harmful to human body. The research of Schade et al. [28] showed that animal husbandry, marine sources and biomass combustion are the main contribution sources of methylamines, while sewage, industrial emissions and automobile exhaust contribute less.

Although OAs have lately received great attention in recent years, due to the low abundance of OA in the atmosphere and the constraints of measurement technology, there is still a lack of understanding of their temporal and spatial distribution, sources and sinks in the atmosphere.

The South to Water North Water Transfer projects originates from Danjiangkou Reservoir in Nanyang City, Henan Province, China. It is a large-scale inter-basin water transfer project. Danjiangkou Reservoir has become a strategic water source to alleviate the shortage of water resources in North China and promote regional coordinated development [29]. The water transmission trunk line of the middle route of the South-to-North Water Transfer Project has a total length of 1432 km, and the annual water transfer is planned to be 9 billion m^3^. It provides domestic, industrial and agricultural water to 19 large and medium-sized cities and more than 100 counties in the North China Plain, including Beijing and Tianjin [30]. The beneficiary population exceeds 200 million, making a great contribution to the social development of the water-receiving area. The water environmental quality of the headworks is directly related to the water safety and quality of the water receiving area. Therefore, the study on the ecological security of the water source area of the water transfer project is very significant to ensure the ecological construction and water supply security of the water source area and realize the ecological security and sustainable development of the water transfer area.

The water quality of the small Pacific water area (hereinafter referred to as Xichuan reservoir area), exceeds the total nitrogen limits, which is reflected in the national monthly report on surface water quality in recent years. The water quality is in a good quality as class III or class II without nitrogen evaluation, but the deterioration of water quality to class IV when introduced in the total nitrogen in the evaluation approach [31].

In order to further reveal the composition of about 50% soluble organic nitrogen besides urea and amino acids, the concentrations and contents of five fatty amines, one heterocyclic amine and five aromatic amines in the atmospheric whole particle sampling samples from October 2020 to November 2021 were determined by the combination of field observation, field investigation and indoor and GC-MS analysis. The contribution rate of 10 kinds of OA to organic nitrogen was evaluated, the component characteristics of soluble organic nitrogen were further clarified, and the main sources of OA were clarified based on the regional concentration distribution law of OA, which provided data support for further research on the environmental behavior of OA in the atmosphere, and provided support for water-quality maintenance and environmental protection of strategic water sources.

## 2. Materials and Methods

### 2.1. Sampling Point Locations and Functional Areas

**Figure 1:**
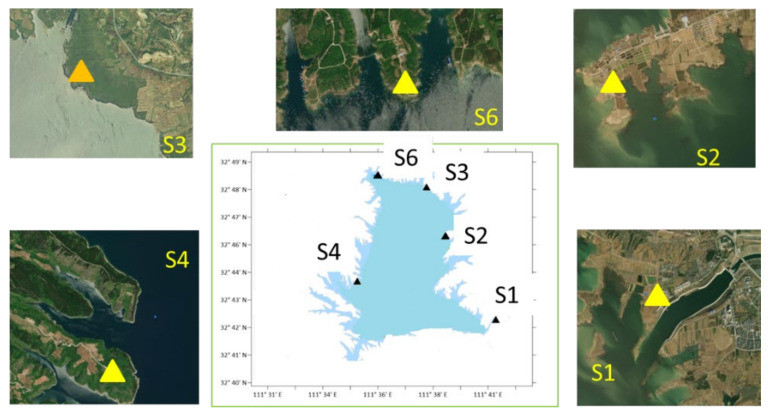
Sampling point position and automatic sampler.

Five sampling points are set around the Xichuan reservoir area of Danjiangkou reservoir, as shown in Figure 1, namely Taocha (S1), Songgang (S2), Tumen (S3), Dangzikou (S4), and Daguanyuan (S6). Atmospheric nitrogen deposition samples are collected and analyzed to determine DTN, dissolved NH_4_^+^-N and NO_3_^−^-N. The DON deposition flux cannot be detected directly, which can be obtained by DON = DTN-NH_4_^+^-N-NO_3_^−^-N.

The automatic samplers were placed on the vary functional area. A sensor was installed on the sampler to control the collection of deposition and wet deposition samples automatically. The machine will automatically open the cover to collect wet deposition once it has rained. The cover will be automatically closed within five minutes after the precipitation.

Standard sample information: there are 10 kinds of standard samples, including 5 kinds of fatty amines, including DMA (10 mg/mL methanol solution), PA, DEA, BA and DBA; 1 kind of heterocyclic amine, PYR, and 4 kinds of aromatic amines, including NMA, BMA, 2-ELA and 4-ELA.

### 2.2. Sample Collection

**Figure 2:**
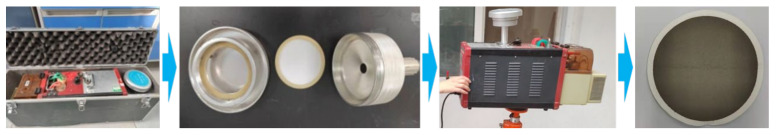
Sample collection method.

The sampler brand is Qingdao Laoying 2021 TSP sampler, and the calibrated flow before sampling is 100 L/min. The filter membrane brand is Whatman quartz microfiber filters. Bake at 500 °C for 4 h before use. The constant temperature and humidity box (the temperature is 25 °C, and the relative humidity is 50%) are balanced for 24 h and then weighed. Samples are stored at low temperature for analysis, as shown in Figure 2.

In this study, full particle samples were collected at five field monitoring stations in the Xichuan Reservoir area from October 2020 to November 2021, and each sample was collected continuously for 48 h; the locations of each monitoring stations are shown in Figure 1.

### 2.3. Sample Pretreatment

**Figure 3:**
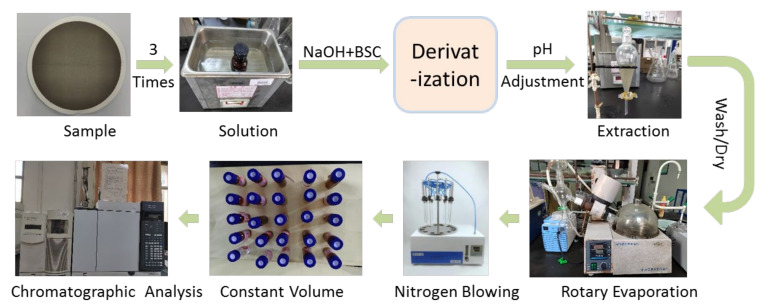
Sample pretreatment process.

Cut 1/4 or 1/2 of the sampling filter membrane and place it in a brown bottle. Add 20 mL of ultrapure water for 15–20 min. Repeat this process 3–4 times. Combine the ultrasonic solution into a flat bottom flask, add about 4 mL of 10 mol/L NaOH solution and 1 mL of benzene sulfonyl chloride (BSC), seal it and stir it magnetically at room temperature for 30 min. Add 5 mL of 10 mol/L NaOH solution to the flat-bottom flask, seal and stir magnetically at 80 °C for 30 min. Place the flat bottom flask in ice water, slowly cool it to room temperature, transfer the solution to the separating funnel, and adjust it to pH = 5.5 with 36.5% HCl. The organic phase was extracted with 10 mL of dichloromethane and collected in a pear-shaped bottle. Wash the organic phase with 10 mL of 0.05 mol/L sodium carbonate solution, dry it with anhydrous sodium sulfate roasted at high temperature, then rotate and evaporate in vacuum at about 40 °C for 3–5 min to about 1 mL, transfer it to the dissecting flask, blow dry or retain about 0.1 mL of the sample under nitrogen flow with a nitrogen blower, and fix the volume with n-hexane to a specific volume (1 or 2 mL according to the actual needs). The whole pretreatment process takes about 1H. GC-MS analysis can then be performed. The sample pretreatment process is as shown in Figure 3.

### 2.4. Preparation of Standard Sample Solution

Firstly, 10 kinds of OA standard samples were prepared into mixed standard sample (100 μg/mL) with methanol, then 1–2 mL was taken for derivatization, and the derivatized solution was finally fixed to 1–2 mL constant volume. The standard sample after derivatization was diluted into different concentration gradients, which were 0.05, 0.1, 0.2, 0.5, 1.0, 2.0, 5.0, 8.0, 10, 15 and 20 μg/mL, respectively.

### 2.5. Chromatographic Conditions

The main contents include: 7890A-5979C gas chromatography-mass spectrometer, DB-5MS chromatographic column; Temperature rise procedure of chromatographic column: 80 °C (1 min), 5 ℃/min → 180 °C, 10 °C/min → 240 °C, 25 °C/min → 290 °C (10 min); Injection port temperature: 290 °C, gas mass transmission line temperature: 290 °C; Carrier gas: high purity helium; Flow: 1.56 mL/min; injection mode: no split injection; mass spectrometry ion source: electron bombardment source (EI, 70 ev); Mass spectrum scanning quality range: *m*/*z* 50~*m*/*z* 450.

## 3. Results

### 3.1. OA Types and Concentration

The concentrations of total nitrogen, organic nitrogen and OA in the atmosphere from December 2020 to November 2021 were detected. The concentrations of ten OA in the atmosphere of the study area in recent one year were measured to provide a basis for analyzing the impact of human activities.

**Figure 4:**
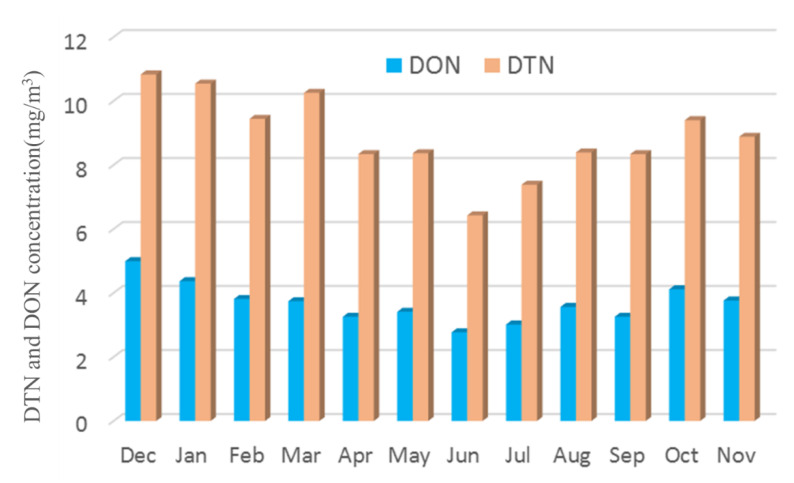
DTN and DON concentration (December 2020–November 2021).

The concentration range of total nitrogen is 6.42–10.82 mg/m^3^, and the concentration of DON is 2.77–4.99 mg/m^3^. The annual average concentrations of DMA, DEA, PA, BA, PYR, N-DBA, NMA, 2-ELA, BMA and 4-ELA were 7.64, 26.35, 14.51, 14.10, 18.55, 7.92, 10.56, 12.84, 13.46 and 21.00 ng/m^3^, respectively, as shown in Figure 4.

**Figure 5:**
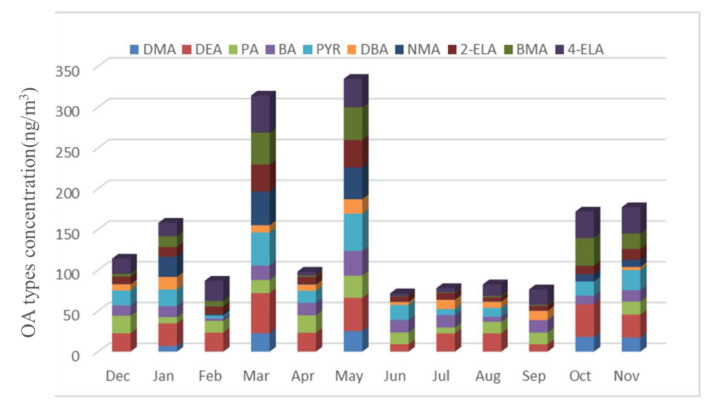
OA types and concentration (December 2020–November 2021).

As shown in Figure 5, The average concentration of ten types of OA reaches the highest value 24.89 ng/m^3^ in spring, followed by 14.17 ng/m^3^ in autumn, 11.97 ng/m^3^ in winter and 7.73 ng/m^3^ in summer.

The sources of OA have significant seasonal differences. The rainfall in spring and autumn is small, the windy weather is frequent, and the proportion of road dust sources is relatively high. Moreover, because spring and autumn are the time of frequent agricultural activities, the concentration of OA is significantly higher than that in winter and summer. The rainfall in summer is large, and the particles in the air are washed by rain, resulting in the lowest concentration of OA in the sample.

The average concentration of ten types of OA reaches the highest value 24.89 ng/m^3^ in spring, followed by 14.17 ng/m^3^ in autumn, 11.97 ng/m^3^ in winter and 7.73 ng/m^3^ in summer.

The temporal and spatial variation characteristics of 10 types of OA are discussed below.

### 3.2. Characteristics of DMA

**Figure 6:**
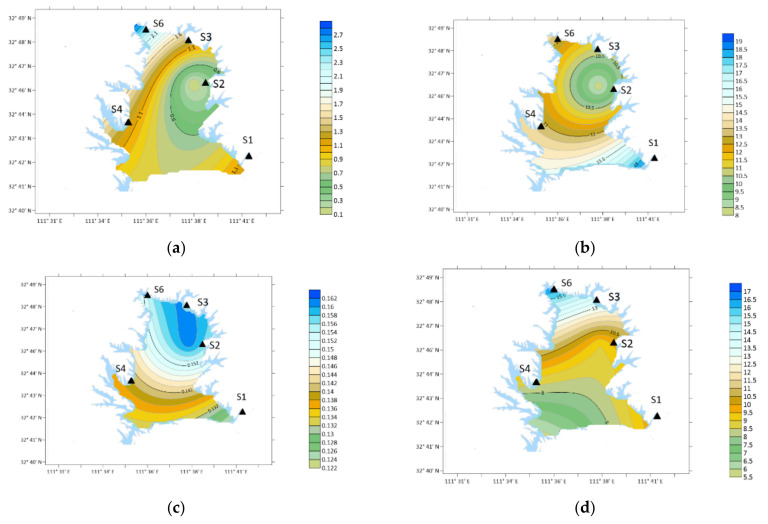
Spatial variation of DMA concentration. (**a**) Winter; (**b**) spring; (**c**) summer; (**d**) autumn.

From the perspective of time, the annual average concentration of DMA was 7.64 ng/m^3^ from December 2020 to November 2021. The concentration of DMA in the atmosphere was high in March, May, October and November, which were 22.57, 25.29, 18.25 and 17.33 ng/m^3^, respectively, as shown in Figure 6.

Spatially, the concentration of DMA in winter: S4 > S6 > S3 > S1 > S2; Spring: S1 > S4 > S6 > S3 > S2; Summer: S2 > S3 > S6 > S4 > S1; Autumn: S4 > S3 > S1 > S2 > S6.

The concentration of DMA was the highest in S4 (orange yard) in the southwest in winter and autumn. In spring, the concentration was the highest at S1 (Urban Area). In summer, the concentration of S2 (Industrial Zone) was the largest position.

### 3.3. Characteristics of DEA

**Figure 7:**
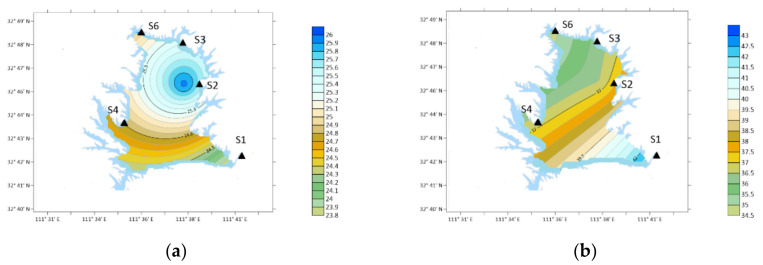

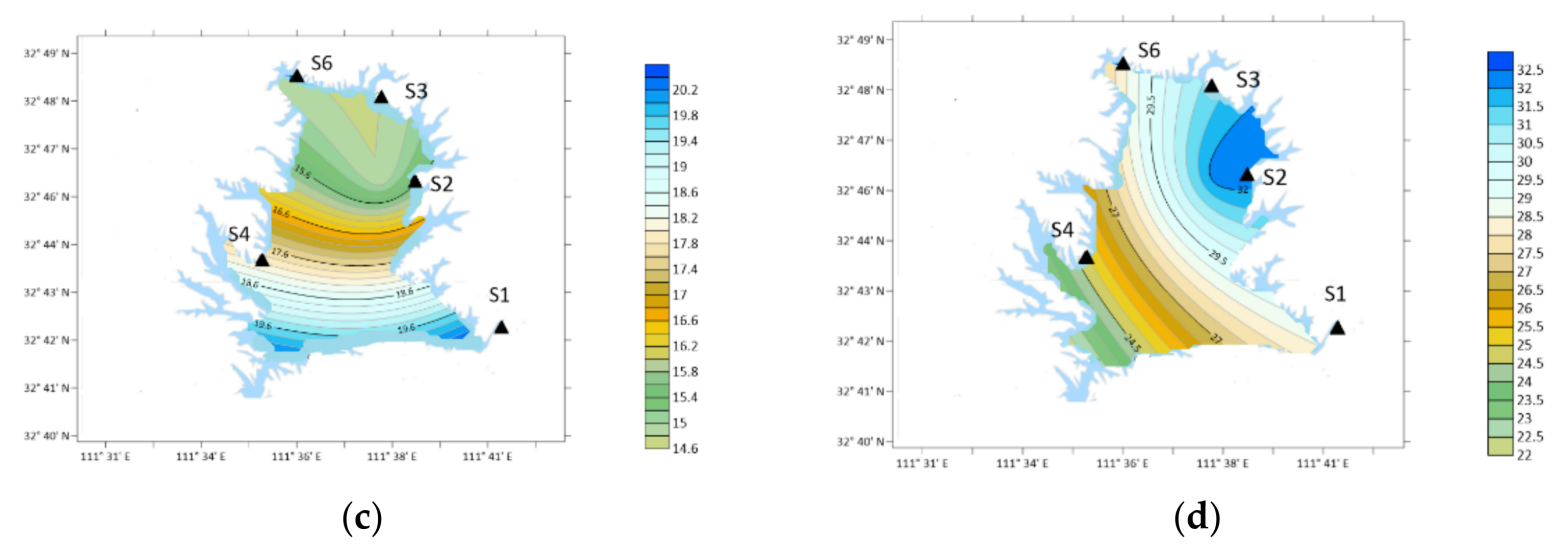
Spatial variation of DEA concentration. (**a**) Winter; (**b**) spring; (**c**) summer; (**d**) autumn.

The annual average concentration of DEA was 26.35 ng/m^3^. The concentration of DEA in the atmosphere was higher in March, May and October, which were 49.23, 40.73 and 39.75 ng/m^3^, respectively, as shown in Figure 7.

Spatially, in winter: S2 > S3 > S4 > S6 > S1; Spring: S1 > S6 > S2 > S3 > S4; Summer: S6 > S1 > S2 > S4 = S3; Autumn: S2 > S3 > S1 > S4 > S6.

The concentration of DEA was the highest in S2 (Industrial Zone) in the southwest in winter and autumn. In spring, the concentration was the highest at S1 (Urban Area). In summer, the concentration of S6 (Forest Area) was the largest position.

### 3.4. Characteristics of PA

**Figure 8:**
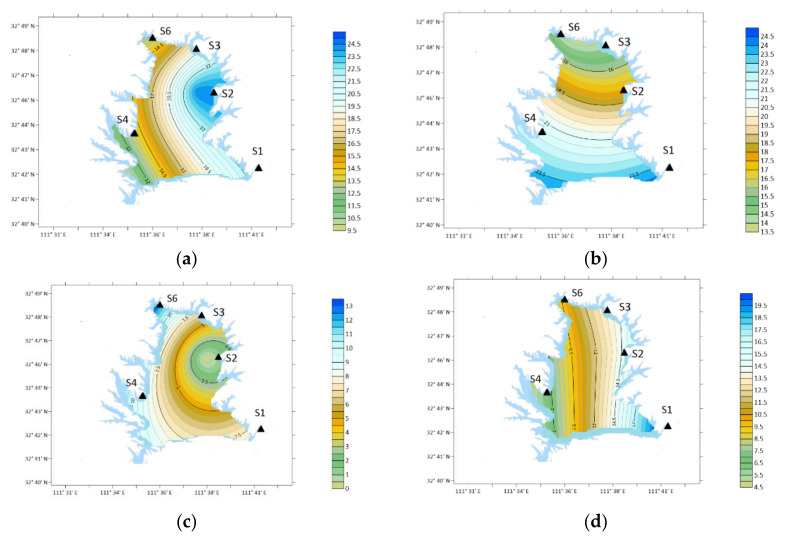
Spatial variation of PA concentration. (**a**) Winter; (**b**) spring; (**c**) summer; (**d**) autumn.

The annual average concentration of PA was 14.51 ng/m^3^. The concentration of PA in the atmosphere was higher in December of 2020 and April and May of 2021, which were 21.47, 21.41 and 27.28 ng/m^3^, respectively, as shown in Figure 8.

Spatially, in winter: S2 > S1 > S3 = S4 > S6; Spring: S1 > S6 > S4 > S2 > S3; Summer: S4 > S1 = S6 > S3 > S2; Autumn: S1 > S2 > S3 > S4 = S6.

The concentration of PA was the highest in S2 (Industrial Area) in the southwest in winter. In spring and autumn, the concentration was the highest at S1 (Urban Area). In summer, the concentration was the highest at S4 (Orange yard) position.

### 3.5. Characteristics of BA

**Figure 9:**
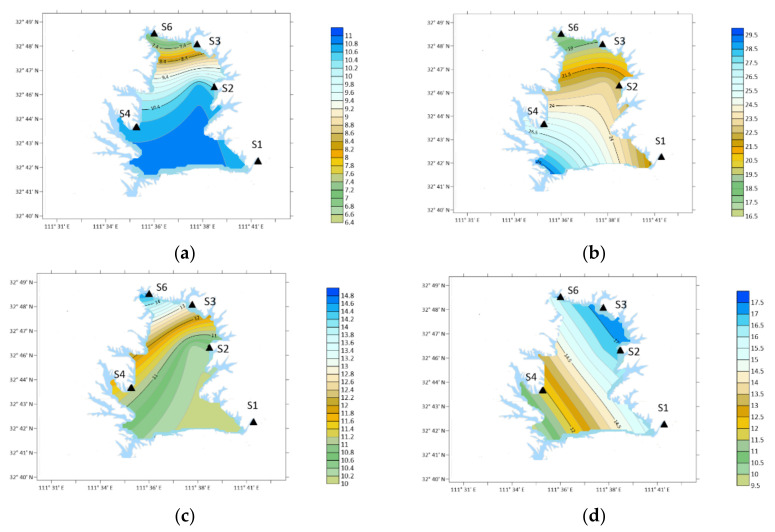
Spatial variation of BA concentration. (**a**) Winter; (**b**) spring; (**c**) summer; (**d**) autumn.

From December 2020 to November 2021, the annual average concentration of BA was 14.10 ng/m^3^. The concentration of BA in the atmosphere was only higher in May, with a concentration of 30.33 ng/m^3^, as shown in Figure 9.

Spatially, in winter: S4 > S6 > S2 > S1 > S3; Spring: S6 > S2 > S1 > S3 > S4; Summer: S4 > S3 > S6 > S2 > S1; Autumn: S1 > S3 > S2 > S4 > S6.

The concentration of BA was the highest in S4 (Orange yard) in the southwest in winter and summer. In spring, the concentration was the highest at S6 (Forest Area). In autumn, the concentration was the highest at S1 (Urban Area).

### 3.6. Characteristics of PYR

**Figure 10:**
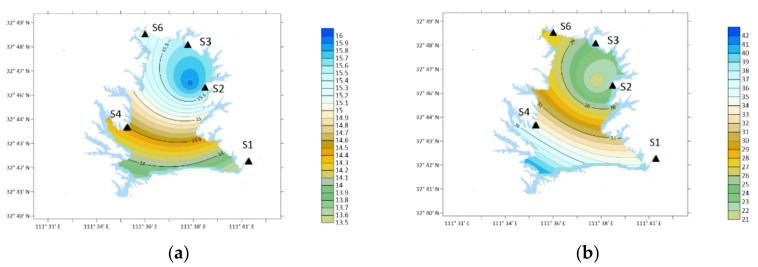

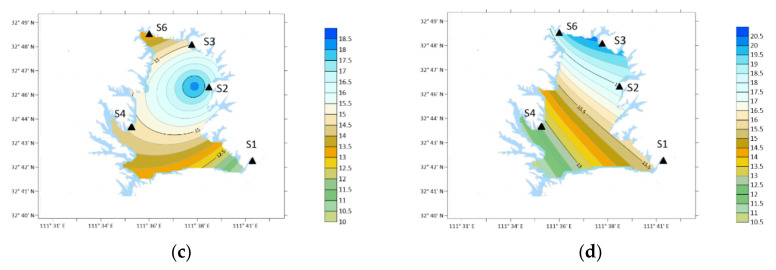
Spatial variation of PYR concentration. (**a**) Winter; (**b**) spring; (**c**) summer; (**d**) autumn.

From the perspective of time, the annual average concentration of PYR was 18.55 ng/m^3^, which was higher in March and May, 40.77 ng/m^3^ and 45.55 ng/m^3^, respectively, as shown in Figure 10.

Spatially, in winter: S2 > S3 > S4 > S6 > S1; Spring: S6 > S1 > S3 > S4 > S2; Summer: S2 > S4 > S3 > S6 > S1; Autumn: S3 > S2 > S1 > S4 > S6.

PYR concentration was the highest in winter and summer and at S2 (Industrial Zone) in the southwest. In spring, the concentration was the highest at S6 (Forest Area). The concentration was the highest in S3 (Agricultural Area) in autumn.

### 3.7. Characteristics of DBA

**Figure 11:**
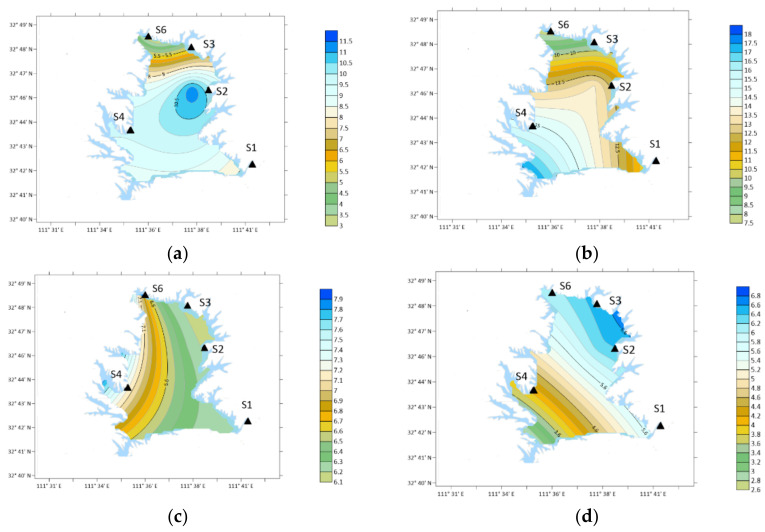
Spatial variation of DBA concentration. (**a**) Winter; (**b**) spring; (**c**) summer; (**d**) autumn.

From the perspective of time, the annual average concentration of DBA was 7.92 ng/m^3^, which was higher in February, May, July and September, which were 15.50, 17.78, 11.38 and 11.36 ng/m^3^, respectively, as shown in Figure 11.

Spatially, in winter: S4 > S2 > S6 > S1 > S3; Spring: S6 > S2 > S4 > S1 > S3; Summer: S4 > S1 = S6 > S2 > S3; Autumn: S1 > S2 > S3 > S4 > S6.

The concentration of DBA was the highest in S4 (Orange yard) in the southwest in winter and summer. In spring, the concentration was the highest at S6 (Forest Area). In autumn, the concentration was the highest at S1 (Urban Area).

### 3.8. Characteristics of NMA

**Figure 12:**
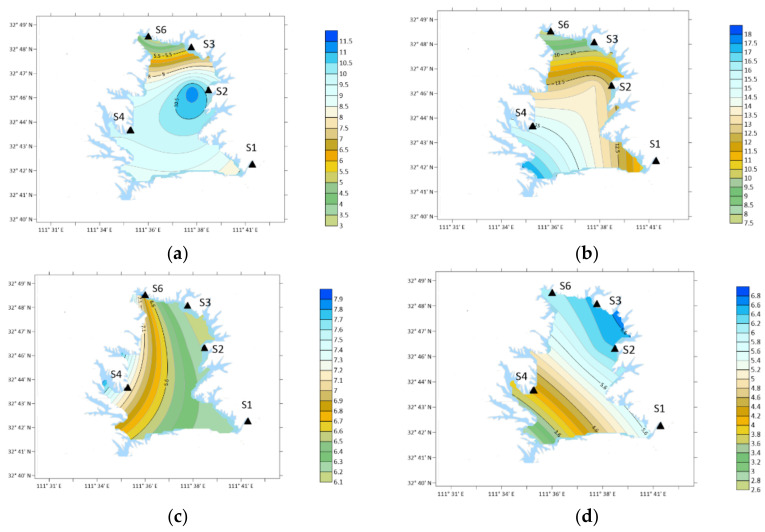
Spatial variation of NMA concentration. (**a**) Winter; (**b**) spring; (**c**) summer; (**d**) autumn.

From the perspective of time, the annual average concentration of NMA was 10.56 ng/m^3^, which was higher in January, March and May, 24.74, 41.18 and 38.70 ng/m^3^, respectively, as shown in Figure 12.

Spatially, in winter: S2 > S6 > S3 > S4 > S1; Spring: S6 > S1 > S4 > S3 > S2; Summer: S6 > S4 > S1 > S3 > S2; Autumn: S3 > S2 > S1 > S4 = S6.

The concentration of NMA is the highest in S2 (Industrial Zone) in the southwest in winter. In spring and summer, the concentration was the highest at S6 (Forest Area). The concentration was the highest in S3 (Agricultural Area) in autumn.

### 3.9. Characteristics of 2-ELA

**Figure 13:**
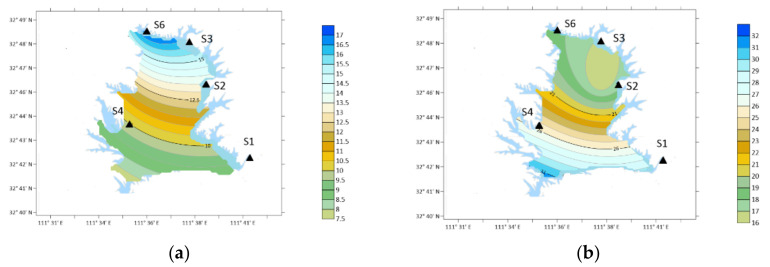

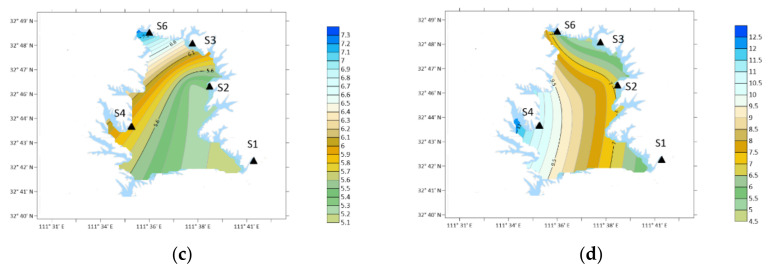
Spatial variation of 2-ELA concentration. (**a**) Winter; (**b**) spring; (**c**) summer; (**d**) autumn.

From the perspective of time, the annual average concentration of 2-ELA was 12.84 ng/m^3^, which was higher in March and May, 33.15 and 33.99 ng/m^3^, respectively, as shown in Figure 13.

Spatially, in winter: S3 > S2 > S1 > S4 > S6. Spring: S6 > S1 > S4 > S3 > S2. Summer: S4 > S3 > S6 > S2 > S1. Autumn: S4 > S6 > S2 > S1 > S3.

The concentration of 2-ELA was the highest in S3 (Agricultural Area) in winter. In spring, the concentration was the highest at S6 (Forest Area). The concentration was the highest at S4 (Orange yard) in summer and autumn.

### 3.10. Characteristics of BMA

**Figure 14:**
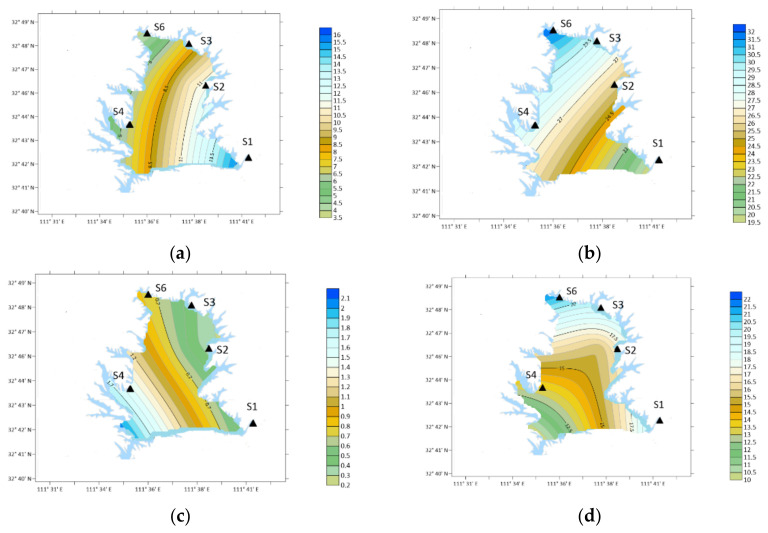
Spatial variation of concentration of benzamine. (**a**) Winter; (**b**) spring; (**c**) summer; (**d**) autumn.

From the perspective of time, the annual average concentration of BMA was 13.46 ng/m^3^, which was higher in March, May and October, 39.14, 40.13 and 34.08 ng/m^3^, respectively, as shown in Figure 14.

Spatially, in winter: S1 > S2 > S3 > S6 > S4; Spring: S4 > S3 > S6 > S2 > S1; Summer: S6 > S4 > S3 > S2 > S1; Autumn: S4 > S1 > S3 > S2 > S6.

The concentration of BMA was the highest in S1 (Urban Area) in winter. In spring and autumn, the concentration was the highest at S4 (Orange yard). In summer, the concentration was the highest at S6 (Forest Area).

### 3.11. Characteristics of 4-ELA

**Figure 15:**
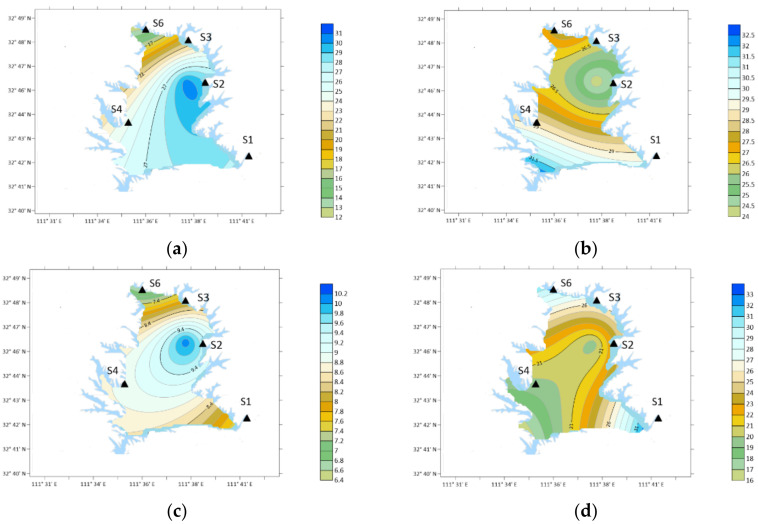
Spatial variation of 4-ELA concentration. (**a**) Winter; (**b**) spring; (**c**) summer; (**d**) autumn.

From the perspective of time, the annual average concentration of 4-ELA was 21.00 ng/m^3^, which was higher in March, May, October and November, 45.59, 34.87, 32.36 and 32.36 ng/m^3^, respectively, as shown in Figure 15.

Spatially, in winter: S2 > S1 > S6 > S3 > S4; Spring: S6 > S1 > S3 > S2 > S4; Summer: S2 > S6 > S4 > S1 > S3; Autumn: S1 > S4 > S3 > S2 > S6.

The concentration of 4-ELA was the highest in S2 (Industrial Zone) in the southwest in winter and summer. In spring, the concentration was the highest at S6 (Forest Area). In autumn, the concentration was the highest at S1 (Urban Area).

## 4. Discussion

In this study, the concentrations of OA in atmospheric particulate matter were compared. The concentrations of methylamine, ethylamine, DMA, trimethylamine and benzenamine in atmospheric particulate matter are about several to tens of 1 ng·m^−3^. The concentrations of 10 types of OA measured in Danjiangkou Reservoir area are within this level, but they belong to the area with high concentration, which is mainly related to the industrial activities in the study area, as shown in Table 1.

OAs come from a variety of sources, mainly including anthropogenic sources and natural sources. In terms of main contribution, natural source is the main source of OA emission, but in large and medium-sized urban areas with dense human distribution, anthropogenic source is an important emission path of OA. At present, the known anthropogenic sources of OA include animal husbandry emissions, industry and combustion, composting, automobile exhaust emissions and human activities.

**Figure 16:**
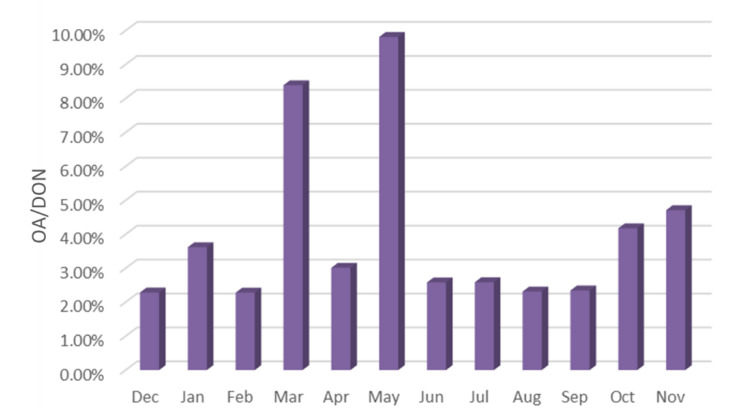
Proportion of OA in DON (December 2020–November 2021).

As shown in Figure 16, the total concentration of ten OA accounted for 2.28–9.81% of DON in the current month, with the highest in March and May, exceeding 8%.

**Figure 17:**
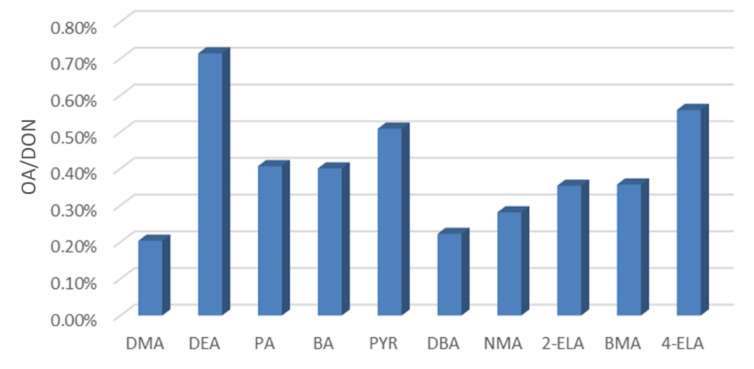
Concentration of ten OA (December 2020–November 2021).

As shown in Figure 17, Among the ten tested OA, the content of DEA is the highest, reaching 0.71%, the contents of 4-ELA, PYR, PA and BA are between 0.4–0.56%, and the contents of DMA, DBA and NMA are between 0.2–0.36%.

As is known to all, NOx is one of the main harmful components of automobile exhaust, and NOx may also lead to the formation of nitrosamines, resulting in the production of amines. In addition, the discharge of urine or wastewater from human activities will become the discharge source of OA. There is evidence that DMA widely exists in human urine and is released into the atmosphere, and hydroxylamine and amide can also be detected in wastewater treatment. DMA has a good correlation with Na and Mg, indicating that DMA mainly comes from primary sources, possibly from microbial degradation in soil and emissions from animal feeding plants. Methylamine has good correlation with Zn, Pb and S. It is speculated that methylamine is the main source of automobile exhaust, coal combustion and industrial emission.

Like the emission principle of animal husbandry, garbage accumulation treatment will produce many anaerobic biological fermentations, which is also an important way to discharge amine. The research shows that nitrogen-containing compounds are an important component of all VOCs produced in the composting process.

The OA ratio and concentration are varying with season. In summer, high temperature, high humidity, strong light and other meteorological conditions cause surface temperature rise, which is suitable for the reproduction of microorganisms in soil and other environments, resulting in the production and volatilization of more OA into the atmosphere.

Ethylamine mainly comes from secondary sources and motor vehicle emissions. DMA mainly comes from biomass combustion, and some comes from motor vehicle emissions and secondary sources. It shows that aniline mainly comes from industrial emission and biomass combustion.

For the emission source of OA in the atmosphere, livestock emission is particularly important. Near livestock facilities, the gas-phase concentration of low molecular weight alkyl amines is as high as hundreds of micrograms per cubic meter. In the atmospheric monitoring of cattle farms, DMA was found to be the main fatty amine. In addition, the study also found that anaerobic bacteria can produce amine through decarboxylation reaction in animal gastrointestinal tract and feces. Therefore, animal husbandry has become an important source of OA in rural areas.

Industrial and combustion emissions of amines include food factory processing, leather processing, fuel and waste combustion, etc. Fish processing is the main emission source of amines in food processing, and the identified amines are mainly fatty amines such as trimethylamine, methylamine, DMA, DEA and PA. Aromatic amines are the main emissions in the leather chemical processing industry. Similarly, polymer combustion and waste combustion will also release some aromatic amines.

In addition, microorganisms contained in the soil can fix N_2_ and convert it into soil nitrogen, and finally discharge it into the atmosphere in the form of ammonia and nitrate. In this process, many nitride products such as methylamine and BMA will volatilize or enter the atmosphere under human influence. Vegetation emission itself is the main source of ammonia, but it may also be the source of amine. Early studies identified OA such as trimethylamine, DEA and methylamine in the air around vegetation.

In addition to the large release of industrial processing, daily cooking and barbecue will also release amines existing in meat or cooked food. In addition, tobacco smoke is also a common emission source of fatty amines. At present, hundreds of nitrogen-containing compounds have been identified in tobacco smoke, including pyrrole, pyridine, pyrazine and some amides in addition to fatty amines and aromatic amines.

## 5. Conclusions

(1) Ten types of OA were detected in the atmospheric particle samples collected in five field monitoring stations around Xichuan Reservoir Area, namely DMA, DEA, PA, BA, PYR, DBA, NMA, 2-ELA, BMA and 4-ELA. The annual average concentrations were 7.64, 26.35, 14.51, 14.10, 18.55, 7.92, 10.56, 12.84, 13.46 and 21.00 ng/m^3^, respectively.
(2) The proportion of OA in organic nitrogen was different, the total nitrogen concentration was 6.42–10.82 mg/m^3^, and the DON concentration was 2.77–4.99 mg/m^3^. The total concentration of ten kinds of OA accounted for 2.28–9.81% of DON in the current month, of which the content of DEA was the highest, reaching 0.71%, the content of 4-ELA, PYR, PA and BA were 0.4–0.56%, and the content of DMA, DBA and NMA was 0.2–0.36%.
(3) The sources of OA in the reservoir area have significant seasonal differences. The content of OA was the highest in spring, followed by autumn, and lower in summer and winter. The rainfall in spring and autumn is small, windy weather is frequent, and the source of road dust is relatively high. Because spring and autumn are the time of frequent agricultural activities, the concentration of OA is significantly higher than that in winter and summer. This shows that agricultural activities have a significant impact on the emission of OA. The rainfall in summer is large, and the particles in the air are washed by rain, resulting in the lowest concentration of OA in the sample.

## Figures and Tables

**Table 1:** Comparison of OA concentration in atmospheric particulate matter.

Place	Sampling Type	Sampling Date (Year~Month)	Concentration/ng·m^−3^					Analytical Method
			MA	EA	DMA	TMA	AN	
Nanjing	Industrial park	12 2017~01 2018	9.6 ± 5	2.6 ± 1.4	12.6 ± 4.8	5.5 ± 2	2.5 ± 1.1	IC
		03 2018~04 2018	12.3 ± 3.9	5.8 ± 1.9	15.3 ± 5.6	7.6 ± 1.6	5.1 ± 1.7	
		07 2018~08 2018	16.9 ± 5.6	10.8 ± 4.6	26.6 ± 14	12.3 ± 3.9	10.6 ± 4.2	
		10 2018~11 2018	14.2 ± 9	6.9 ± 4	22.2 ± 13.6	9.5 ± 4.6	6.3 ± 4.3	
Guangzhou	Mountain area	10 2016	8.79 ± 7.80	-	2.37 ± 3.15	-	-	GC—MS [32]
		05 2017~06 2017	11.9 ± 9.77	-	5.03 ± 2.23	-	-	
Nanjing, Yangzhou	Urban area	11 2015~04 2016	5.7 ± 3.2	20.3 ± 16.6	7.9 ± 5.4	-	-	HPLC [33]
Beijing	Urban area	01 2013~02 2013	31 ± 10.8	14.8 ± 6.5	4.3 ± 1.7	-	5.7 ± 2.2	HPLC [34]
Xi’an	Urban area	01 2013~02 2013	24.7 ± 9.7	12.6 ± 5.6	3.8 ± 1.6	-	5.1 ± 2.2	
Xiamen	Urban area	01 2013~02 2013	10.2 ± 2.0	5.3 ± 1.4	1.7 ± 0.4	-	2.5 ± 0.8	
HongKong, China	Urban area	01 2013~02 2013	12.1 ± 2.4	4.5 ± 1.1	1.5 ± 0.5	-	2.0 ± 0.6	
Eastern China	Ocean	08 2015	-	-	34.2 ± 5.4	55.8 ± 7.8	-	IC [35]
		04 2015	-	-	9.9 ± 5.4	16.8 ± 9.8	-	
Xi’an	Urban area	03 2009~05 2009	16.9 ± 5.5	9.7 ± 3.9	-	-	-	HPLC [36]
		06 2009~08 2009	6.2 ± 2	3.8 ± 1.4	-	-	-	
		09 2008~11 2008	14.7 ± 9.9	8.4 ± 4.2	-	-	-	
		12 2008~02 2009	22.3 ± 10.4	11.5 ± 4.1	-	-	-	
Zonguldak Province, Turkey	Ocean	10 2006~04 2007	4.48 ± 1.75	4.37 ± 2.27	4.58 ± 2.28	-	5.52 ± 2.05	HPLC [37]
		05 2007~09 2007	2.33 ± 1.30	2.19 ± 1.13	2.79 ± 1.55	-	3.63 ± 2.36	
Tampa Province, USA	Ocean	06 2005~08 2005	-	-	31 ± 28	-	-	IC [38]
Jeju Island, Korea	Ocean	03 2001~04 2001	13.5	3.1	-	-	-	HPLC [39]

## Data Availability

Data available on request.
